# Assessing an integrated team-teaching lecture in medicine and surgery program- Galala University

**DOI:** 10.1186/s12909-024-05685-8

**Published:** 2024-07-12

**Authors:** Ahmed Nour Eldin Hassan, Reem M. Sallam, Sara Kamal Mattout, Noha Nooh Lasheen

**Affiliations:** 1 Faculty of Medicine, Galala University, Suez, Egypt; 2 Faculty of Medicine, Ain Shams University, Cairo, Egypt; 3 Medical Education Researcher, Galala University, Suez, Egypt

**Keywords:** Integration, Co-teaching, Basic medical sciences, Effective medical education, Student-created question, Students’ activity

## Abstract

**Introduction:**

Achieving integration in medical curricula without redundancy in basic medical sciences disciplines is a substantial challenge. Introducing co-teaching in such curricula with active inter-disciplinary participation is believed to best utilize the teaching and learning time for instructors and students, to motivate the students, and to provide a more robust base for bridging the gap between basic and clinical medical sciences in medical schools. Additionally, including more than one student-centered activity in one session is expected to increase the students’ involvement and improve the retention of knowledge. Our study aims at minimizing redundancy and improving the students’ motivation in learning the topic “insulin-glucose regulation” during the Endocrine and Metabolism module taught to year three students at Galala University, Faculty of Medicine in Egypt.

**Methods:**

The authors designed a 3-hr co-teaching integrated session with 3 basic medical sciences aimed to explain the clinical terms including online accessed pre/post-tests, small student groups-created pre/post-session MCQ, with co-sharing of students in the introduction of scientific materials.

**Results:**

The students’ scores in the post-test showed that they gained more knowledge compared to before. Interestingly, there was only an improvement in the students’ performance in generating questions before and after the session, as well as in the integrated question in the end-of-semester exam, we suggest implementing this approach in other topics and modules in medical schools. It would also be favorable to follow up with the students taught using this approach and those taught differently to assess the effectiveness of this approach in a controlled manner.

**Conclusion:**

Integrated sessions effectively increase student awareness of medical concepts and reduce redundancy in basic medical sciences. This approach exposes students to a more comprehensive understanding of the subject matter, improving their comprehension and retention. It is a valuable method for educators and instructors seeking to enhance their students’ learning experience in the field of medical sciences.

## Introduction

Co-teaching is more beneficial for students as it enables multiple teachers to leverage their individual areas of expertise. Curriculum experts can focus on content, while special educators can bring their skills in pedagogy to create a more comprehensive learning experience [1]. Organization of co-teacher instruction can be performed by many models, with varying roles and responsibilities [2]. In the *team-teaching* model, all co-teachers share the lead instructional roles with students and are equally active. In the second model, which is the *alternative* and *parallel* models, the class is divided into two halves (parallel classes), or one large and one small group (alternative); each co-teacher leads instruction in a group. The fourth model is *Station teaching*, which is composed of three stations, with each co-teacher leading one station and a third independent station. In these four co-teaching models, each co-teacher has a role.

On the other hand, one teacher leads instruction, while the other observes, circulates, or assists students in the *one-teach/one-assist* and *one-teach/one-observe* models, depending on the learning needs of students [3]. The role of the assisting or observing teacher has been described as the support role [4, 5]. This model is considered the most widely used co-teaching model [6, 7].

In co-taught classes, students have shown positive academic gains across multiple subject areas [1, 8, 9]. However, some studies found no difference in effectiveness compared to solo-taught classes [10, 11].

Further, it has been discovered that within medical faculties around the world, curricular redundancy is a notable problem, especially in those with a highly integrated curriculum [12]. Despite the need for some redundancy to reinforce certain information, it better be at a minimum to ensure the best utilization of teaching and learning time. Efficient time utilization is particularly crucial in basic medical sciences (BMSs) disciplines taught in medicine programs. This is especially true given the recent shortening of student years in Egypt in 2017. This substantial reform has been approved and implemented nationwide by the Supreme Council of Egyptian Universities (SCU). The reform included changing the timeline of all medical programs from six years of studentship plus one-year clerkship to five years plus two years, respectively [13]. Moreover, the reform called for replacing discipline-based curricula with a modular integrated model, emphasizing early clinical exposure. In faculties of Medicine in Egypt, the first half of the 5-year-studentship duration comprises preclinical integrated modules expected to provide the students with the required medical knowledge, skills, and competencies relevant to various body systems in an interdisciplinary approach [14]. One of the primary challenges during curricular development is poor inter-disciplinary communication among teaching instructors and lack of concept of integration among the students [15]; resistance to change in medical curricula in most faculties has further imposed another difficulty. These problems were highly noticed when dealing with a relatively large number of students, which raised the lack of proper active participation of students in the learning process.

In recent years, active learning has gained a lot of interest among the medical education communities. During active learning, knowledge is acquired and synthesized through student-led activities. One active learning method shown to be effective for teaching is question creation, which involves students actively participating in the formulation of questions. Such student-centered activity promotes critical thinking and enhances students’ understanding of complex medical concepts. According to a study conducted by Heitz and coworkers, engaging students in question creation not only improves their knowledge retention but also fosters a deeper understanding of the subject matter. Furthermore, this activity encourages students to think from different perspectives and develop adequate communication skills. Through actively participating in question creation, students become more engaged in the learning process and take ownership of their education. This leads to better academic performance and long-term knowledge retention [16], despite being claimed to be time-consuming and requiring more effort [17].

Integrating basic medical sciences (BMSs) with clinical concepts in medical schools has always been a critical challenge for medical education. Various educational strategies have been experimented with and/or adopted for reaching the required integration during undergraduate medical years at multiple levels of the curriculum. Documented approaches to integration at the level of programs, courses/modules, or teaching sessions aimed at improvement of learning outcomes [1]. Further challenges faced in executing general practitioner medical graduates are curricular redundancy and the lack of clear goals regarding the required extent of knowledge.

Integrated learning empowers learners to efficiently acquire, assess, prioritize knowledge according to importance, and filter out irrelevant information [18]. By adopting this approach, learners can maximize their cognitive potential to comprehend knowledge with ease and efficiency.

### Aim of the work

The purpose of this study is to introduce a *team-teaching* model to reduce redundancy within an integrated curriculum at a newly established university. The proposed *team-teaching* model further aspires to enrich the students’ understanding and reach higher cognitive levels of the taught topic, through active involvement of students in the delivery of materials. Our strategy involved presenting the topic of regulation of glucose by insulin in a multidisciplinary manner and allowing students to formulate relevant integrated questions. This study was conducted during the Endocrine and Metabolism module taught to third-year medical students in the academic year 2022–2023 at Galala University, one of the newly established universities in Egypt. The authors of this work are the module coordinator (Ahmed Nour Eldin Hassan) and the program director (Noha N. Lasheen). The instructors for the participating BMSs’ disciplines are Biochemistry (Reem M. Sallam), Physiology (N.N.L.), and Pharmacology (A.N.H.); and a medical education specialist (S.K.M.).

In addition, to test the concept of integration to the students, they were asked to formulate one multiple choice question before and after a session on the same topic.

We aimed to answer the following research questions:

- Can the integration and knowledge-acquiring process of students in the glucose regulation by insulin-topic be improved by reducing the redundancy of BMSs and by sharing the delivery of materials?
- Will this improvement be reflected in their performance in a pre-and post-test, and in their ability to create questions more efficiently?
- Does the involvement of an integrated case in the end-of-semester exam improve student engagement in the learning process?

## Methods

### Participants

All third-year students in the Program of Medicine and Surgery at Galala University (*n* = 104 students) participated in this study during the Endocrine and Metabolism Module (course code: BMS303). The limited module duration (3 weeks) triggered the module coordinator and participating instructors to apply an innovative approach for the best utilization of time, and potentially better students’ comprehension of the topic of choice: insulin-glucose regulation. The integration of the three BMS disciplines in one session was expected to reduce redundancy compared to teaching the topic separately by each discipline, which would take up to 6 h of lecture teaching. Throughout the integrated session, BMS’s reasoning and background on various clinical situations relevant to the topic of interest were emphasized. The study was performed in December 2022, three weeks prior to the students’ end-of-semester exams.

### Procedure

The process started with the module coordinator revising a brief course contents file previously prepared by the Scientific Committee of the Program of Medicine and Surgery at Galala University. This was followed by calling for an online meeting involving the co-authors of the current work. During the meeting, the participants aimed to map out the curriculum for a specific module. This involved identifying any gaps or overlaps in the available brief file and agreeing on a topic to be taught through horizontal-dimension integration between the participating BMSs’ disciplines. The topic of insulin-glucose regulation was chosen to be prepared and presented in an interdisciplinary approach. An interactive discussion of the topic’s contents was held in the same meeting, with participation from the disciplines of Medical Biochemistry, Medical Physiology, and Clinical Pharmacology.

Later, the module coordinator collected PowerPoint slides from BMS instructors and created an integrated PowerPoint presentation (PPT) that outlines session contents, intended learning outcomes (ILOs), and timeline. PPT core includes slides from each discipline, eliminating overlaps and rearranging the slide sequence for interdisciplinary integration. PPT was circulated among the co-authors for revision and recommendations. During a second online meeting, an updated version of the PPT was shared. The design flow of the session was agreed upon, and a case vignette for a patient with diabetes mellitus was created. Instructors from each BMS discipline were asked to provide 2–3 multiple-choice questions (MCQ) that are relevant to the vignette. The questions will be used as a summative assessment in the end-of-semester exam, in addition to 2 “different” MCQs/disciplines for the pre-and post-tests.

One week before the scheduled integrated session, the module coordinator sent a detailed announcement to third-year students on Canvas, which is the Learning Management System (LMS) platform used at Galala University. The announcement explained the flow of the session, highlighted the intended learning outcomes (ILOs), and emphasized the importance of this approach. The coordinator encouraged the students to attend and utilize the dedicated time to the best of their abilities. The students did the prestest at the start of the team-teaching lecture and neither they were requested to have a previous search of the studied topic before the lecture, nor it was a flipped classroom.

The interdisciplinary session was designed to span three hours. The module coordinator started the session with a 10-minute introduction, during which information about the session logistics was shared and two important announcements were made. First, the session is interactive; hence, all actively participating attending students will earn maximum grades for the course assignment and coursework (15 marks out of a total of 150 marks for the module). Second, the topic would be assessed in the end-of-semester exam as an integrated case with questions from all BMSs’ disciplines participating in the session.

Afterwards, students completed a 10-minute electronic pre-test on the Canvas platform consisting of six MCQs. This pre-test assessed students’ prior knowledge, if any, regarding the three BMSs disciplines with equal weighting: two MCQs/discipline. The same MCQs (post-test) were performed by students at the end of the integrated lecture.

After the pre-test, a three-phase question-creation activity was conducted. In the first phase, instructions phase, students were provided with general instructions on the basics of creating an integrated MCQ and formulating four or five suitable choices. This phase spanned 10 min and aimed to enhance the students’ understanding of the process of question construction. In the second phase, the pre-session question creation phase, students were randomized into small groups (5–6 students/group), and were instructed to create a pre-session integrated question related to the main topic. Additionally, they formulated suitable distractors under the supervision of the instructors. The allocated time for this phase was 10 min. The third phase, known as the post-session question creation phase, involved students spending 10 min formulating a second set of questions based on their comprehension of the integrated collaborative session. During this 3rd phase, the instructors were actively navigating between the students’ small groups providing them brief feedback on their performance in MCQ creation. Students who did not receive feedback during the session were advised to meet with their instructors afterwards.

The core session was conducted at the end of the pre-session integrated question creation phase for 2 h as joint teaching by the instructors. The session was moderated by the course coordinator (A.N.H.), who engaged the students by asking thought-provoking questions, allowing them to collaborate in groups to research specific medical websites for answers, and then discuss with other groups.

In the end-of-semester exam of the Endocrine and Metabolism module, the already prepared integrated case (clinical vignettes) together with the MCQs representing each of the three participating BMSs’ disciplines were included. This was according to the module assessment blueprint, the mapping of test items to the module’s ILOs.

### Statistical analyses

Data were collected, revised, and then, subjected to statistical analysis using one-way ANOVA performed by SPSS.21 program (IBM Inc. Chicago, Illinois, USA). For comparison, all scores were expressed as percentages of the correct answers in the pre-test and post-test. In addition, we compared students’ performance on questions related to the integrated case topic with their performance on the rest of the end-of-semester exam. This comparison can be used as an indicator of knowledge acquisition and understanding.

## Results

In this study, 104 Year 3 students participated representing 100% of third-year students in Galala University Medicine and Surgery Program.

**Fig. 1:**
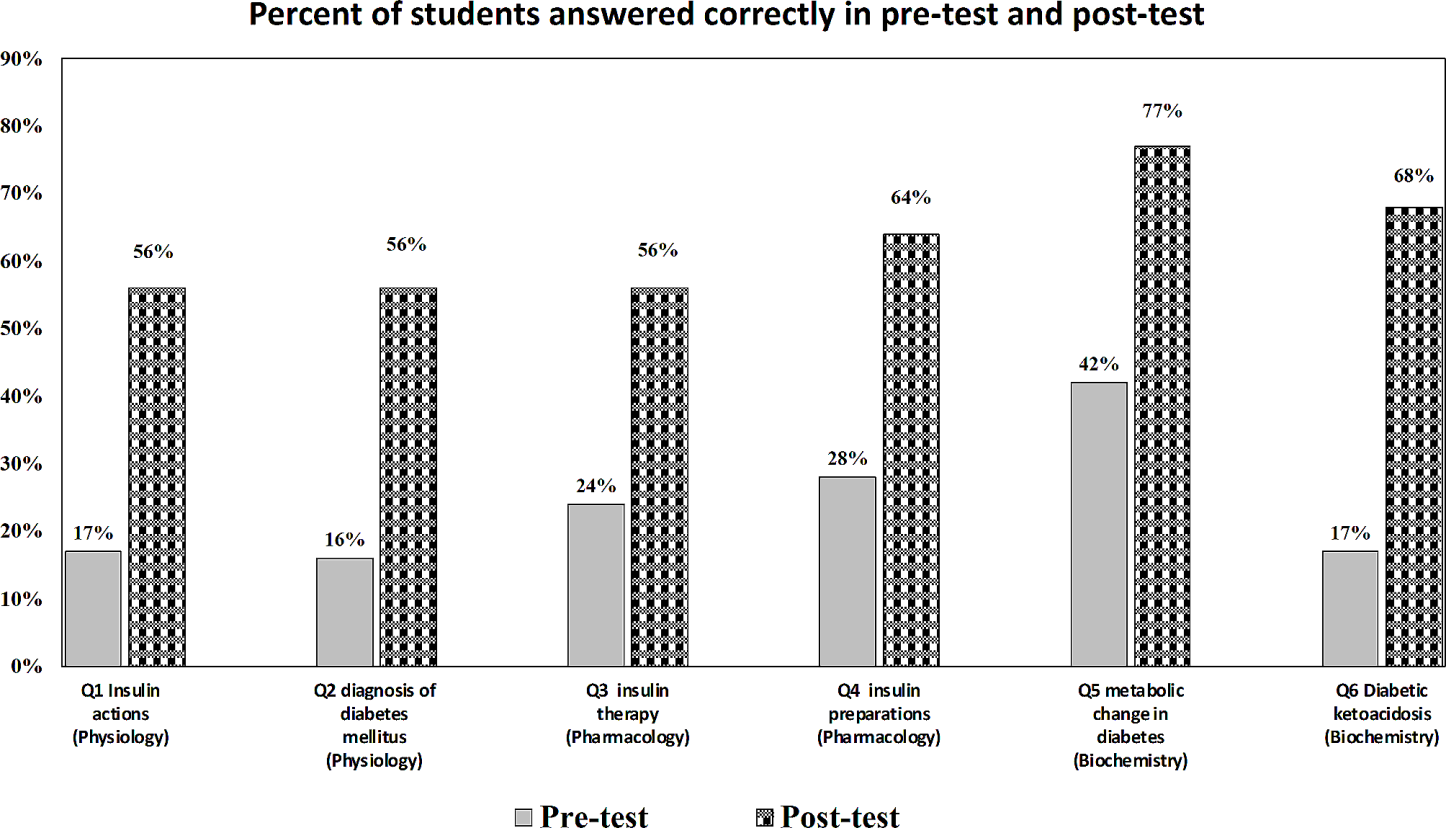
Comparison between the performance of students in pre-and post-test as manifested by the percentage of students who answered each of the six MCQs correctly in pre- and post-tests

The results shown in Fig. 1 indicate an improvement in the students’ test scores in the post-test compared to the pre-test. This was observed in all six questions, where a higher percentage of students answered correctly in the post-test than in the pre-test. Precisely, it was found that the improvement percent was doubled in questions number 3, 4 and 5, tripled in questions number 1 and 2, and quadrupled in question number 6.

**Fig. 2:**
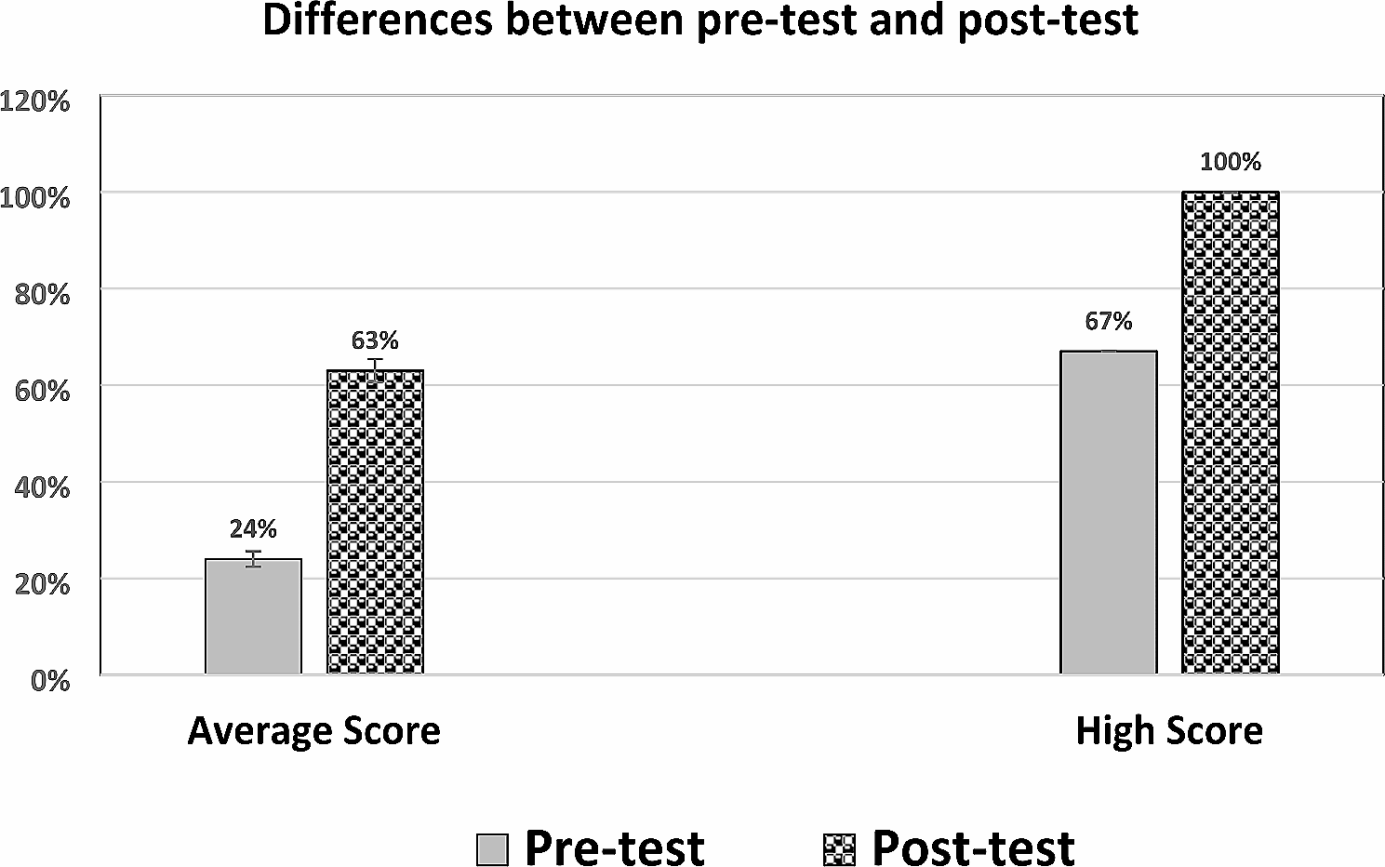
Comparison between the average +/- SD of the percentage of students who answered correctly in pre- and post-tests

Figure 2 shows the collective percentage of students who answered correctly in the pre-test vs. post-test. The results of average ± SD are 24 ± 9.1, and 63 ±7.8 in the pre- vs. post-test, respectively. The highest attained scores in pre- and post-tests were 67% and 100%, respectively. Such changes point to the efficacy of the implemented integrated session on the gain of information by students.

Through analyzing the students’ grades at the end-of-semester exam, there was no statistically significant difference in the students’ performance in the integrated questions vs. their performance in the rest of the exam. The mean ± SD of students percentage answered the integrated questions correctly is 75% ±22.9; while for the rest of the MCQs, the number is 77.5% ± 15.2.

We have applied a simple evaluation tool to assess the MCQs generated by students. This tool briefly evaluates the MCQ by commenting on the question stem, the lead-in question, and the alternatives. It was observed that there was a minor improvement, which might reflect the short instructional period given to students to learn how to formulate good MCQs.

**Fig. 3:**
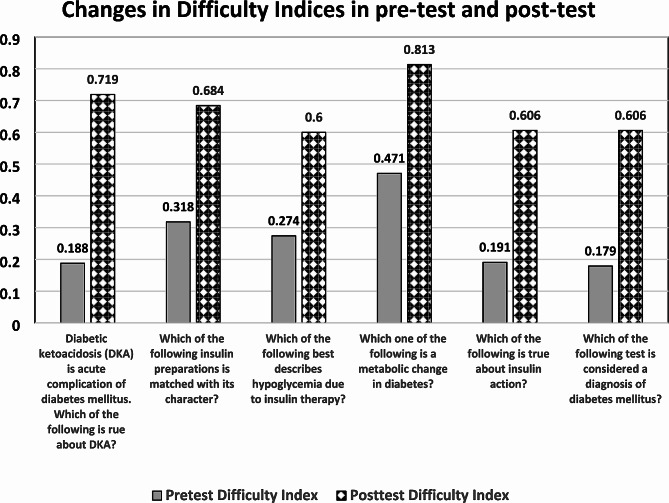
Comparison between difficulty indices in pre-and post-test of answering each of the six MCQs correctly in pre- and post-tests

When comparing the difficulty indices of the same questions in pre- and post-tests, there was a significant rise in post-test, indicating a better understanding and performance of students after completion of the co-teaching integrated session, as shown in Fig. 3. Also, it is noted that only two questions in the pre-test have an acceptable difficulty index of more than 0.3, while the rest of the questions had below-normal difficulty indices. Such a finding denotes the guessing of students in answering. On the other hand, all questions in the post-test had a range of 0.6 to 0.8 difficulty index, which points to the gain of knowledge by students after completion of the session and formulating their integrated questions.

**Fig. 4:**
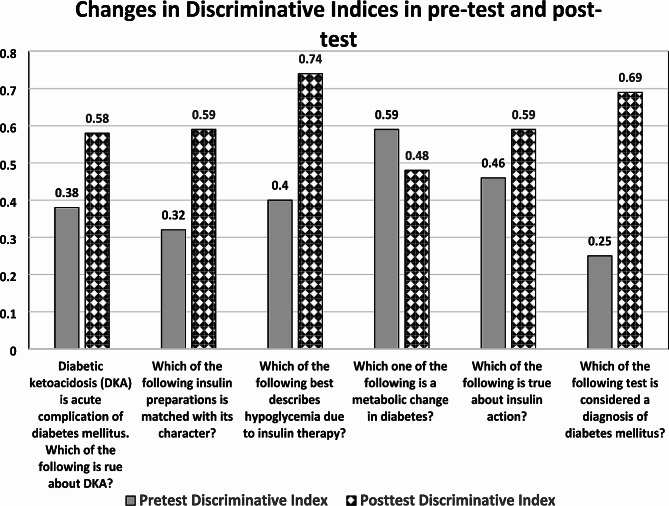
Comparison between discriminative indices in pre-and post-test of answering each of the six MCQs correctly in pre- and post-tests

Regarding discriminative indices of the same used questions in pre- and post-tests, they were significantly elevated in the post-test, compared to the pre-test. All questions have higher values than 0.19, this signifies the positive impact of the session on students ‘performance, as displayed in Fig. 4.

## Discussion

The integrated session described in the current work represented a major feature of the Endocrine and Metabolism module. The session spanned 3 h and included 2 types of activities besides the core teaching session: pre/post-tests, and pre/post-session students-generated questions. In addition, the students have an active role in co-teaching by searching the internet for one related topic and performing peer discussions with colleagues. In our experience, one of the drawbacks of previous integrated lectures is that the students were just listening to the one-way lecture. The concept of integration as perceived by the students was tested by asking each of the small groups of students to construct an integrated question at the beginning of the session, and another one at the end. Analyzing and evaluating the groups’ questions in both time points revealed a minor improvement in students’ performance on the second attempt. This may reflect the short duration for giving instructions by the course coordinator, which was insufficient to train the students on the technicality of creating a good MCQ. However, for peer interaction, and to grasp the extent of knowledge acquired by the students, examples of questions were discussed and answered by students. This exercise has shown a positive and constructive competition attitude between students’ groups.

Various educational strategies exist to fulfil basic and clinical medical sciences integration. In designing the integrated session of insulin-glucose regulation in Endocrine and Metabolism module, shared planning and teaching at the same session were undertaken. At the level of the whole module, we believe that we reached step 8 of Harden Ladder of Integration. As such, the module would be described as a complementary, or mixed module, where both subject-based and integrated teaching were included.

In line, Harden has recently described medical schools, arguing that medical institutions need to move higher up the integration ladder [19], until reaching the top level -step 11 in the Harden Ladder, emphasizing integration in the real-world setting, and revealing that students will start their studies in a clinical setting [20].

It is of value to demonstrate that statistical analysis of the students’ performance in the online accessed pre- and post-tests, composed of identical questions, demonstrated a significant improvement in the knowledge comprehended by the students. This proves that the interactive discussion during the core session between the students and the instructors was successful in targeting several concepts that were not yet clear to the students at the beginning of the session.

A well-planned approach to integrating various disciplines horizontally and utilizing active learning tools with the involvement of students during the preclinical years of the Medical Program resulted in improved knowledge retention. This approach helps students to reason through various clinical situations they are likely to face in the clinical years of medical studies and beyond.

For the assessment of this session, an integrated case-based scenario question was included at the end-of-semester exam assessing the knowledge gained by the students from such an approach. Despite there being no statistically significant difference between the students’ performance in the integrated question relative to their performance in the rest of the exam, we believe that repeating this approach in other modules will probably be needed to document significant improvement.

Numerous studies have consistently demonstrated that student question creation not only facilitates learning but also contributes to personal development. The “depth dynamic model” proposes that answering questions in a contextualized manner can improve deep thinking and reasoning. It suggests that creating questions can encourage hypothesizing, predicting, thought experimenting, and explaining. This can lead to a cascade of generative activity, which helps students acquire missing knowledge or resolve conflicts in their understanding. Learning occurs as cognitive networks which are formed and rearranged through the creation of explanations and answers to each question. According to “information processing theory”, these procedures promote deeper information processing, improve learning outcomes, and achieve higher levels of cognitive development [21]. A previously published study investigated the effects of question-creating activities on students’ learning outcomes and highlighted that creating their own questions had a significantly better understanding of the material than answering instructor-generated questions. The authors of this study concluded that question-creating activities can help students develop critical thinking skills and improve communication skills [22]. In support, another study suggested that this method is more effective for acquiring materials compared to additional studying of the subjects, they mentioned that it had an impact on the learning process and the long-term retention of knowledge among medical students [23]. The students’ engagement in generating questions was, also, described as an effective method for enhancing the students’ academic performance and fostering their motivation [24].

Because formulating the questions in the same topic was performed at the beginning and at the end of the session, we were able to emphasize the concept of teamwork and collaboration between the students; and also, to follow up on their understanding and comprehension progression after attending the session. That is why we could refer to it as “innovation’’.

Within the module’s schedule, two related sessions were positioned following the described integrated session, aiming at building upon what has been provided in the integrated session. The first was conducted by the clinical pharmacology discipline and focused on further pharmacological intervention in cases of diabetes and diabetes complications.

As regards clinical pharmacology, other basic medical sciences background is needed to build upon it and respond to several types of questions frequently asked by medical students. For instance: What is the appropriate sequence of intervention medication tackling cases of emergencies in diabetes? Why should we use insulin in small doses by intravenous infusion in an emergency as diabetic ketoacidosis? What is the biochemical, pathophysiological, and hence management difference between diabetic ketoacidosis and hyperosmolar coma? What is the best choice of insulin type in the management of diabetes? All these questions, and more, were explained on biochemical and physiological bases to provide logic to the pharmacological intervention.

The second was conducted by the instructor of clinical medical sciences and focused on actual case-based scenarios emphasizing their clinical management. Both sessions, although conducted as a single-subject-based teaching, benefited from the prior BMS’s knowledge gained by the students through the described integrated session, as stated by the instructors’ feedback (personal communication with the instructors of both sessions). Instructors’ feedback demonstrated positive remarks on the outcomes of the integrated session, which helped them in best utilizing their own session’s duration in explaining the clinical decision and in introducing some clinical tips and tricks in the management of diabetes, instead of spending the session in providing basic explanation and reasoning for each clinical step.

Thus, it is worth highlighting that creating questions requires students to think critically about the topic and identify key concepts and connections. This process helps the students develop a deeper understanding of insulin-glucose regulation by analyzing the information, formulating questions, and considering potential answers.

From the teaching faculty perspective, participation in well-designed integrated sessions preceded by pre-session professional brainstorming is clearly an advantageous experience that overcomes several communication gaps and overlaps.

Medical education tools could be applied differently based on the context of the learning setting. Similar to the Team Based Learning (TBL), our students’ groups were having the same task to work on, in our case it was the generation of questions. In addition, the objectives and ILOs of our session, as in a TBL session, were distributed to the students prior to the session, albeit no pre-readings were sent to the students as would be the case with a TBL session. In preparing TBL sessions, pre-readings are sent for the students to master prior to attending the session, we did not apply the pre-readings since we aimed to assess the effect of the tool used on the knowledge acquired by the students during the session through pre- and post-tests. Furthermore, TBL involves the students in setting the grading criteria with emphasis on peer evaluation; aspects that were not applied in our model.

The current work has some strength points and other weaker points that need to be improved in further research. Points of strength include the collaborative contribution of participating instructors with students to perform the session to overcome the lack of interest of students in lectures. In addition, the joint teaching of the session by all instructors increased the richness of shared knowledge, and provided the students, indirectly, with a true sense of integration of all medical sciences. Another strength point is its ability to incorporate multiple “relevant” student activities in a single session at different time points. This includes an activity that depends on the students’ creativity, which can enhance their motivation and sense of belongingness and self-satisfaction, in a manner expected to improve their later competencies. The inclusion of an integrated-case question in the end-of-semester exam is believed to encourage the students to exert extra effort to understand the human body in a more holistic approach.

In contrast, this work has some weaker points. Despite the positive feedback comments given by the participating instructors when reporting their experience to the academic committee of the Faculty of Medicine, the single-time nature, and the restricted number of participating instructors in the current work are insufficient to draw quantitative evaluation for the instructors’ feedback. In addition, there were minimal instructions given to the students on how to construct a “good” MCQ at the introductory phase of the session. Therefore, the lack of formal training for the students on this skill may be a confounding factor in assessing the pre- and post-session students-created questions.

In conclusion, the integrated session of “insulin-glucose regulation” in the Endocrine and Metabolism module at the Medicine and Surgery program, at Galala University is considered an effective and appropriate method to bridge the gap between the basic and clinical medical sciences. If well-designed; removing redundancy, and integrating the knowledge, without reducing the ILOs agreed upon are all expected to improve the efficiency of medical topics teaching and learning. More importantly, the students’ sharing in the delivery of materials and in more than one “relevant” student activities in one session increased the students’ motivation, self-satisfaction, and sense of belongingness, and improved their academic performance. The implemented session resulted in better utilization of both the instructors’ and the students’ time. In conclusion, we recommend the replication of the current experience in other topics and in different modules in medical schools.

## Data Availability

Upon request from the corresponding author.
